# Multi-Camera Sensor System for 3D Segmentation and Localization of Multiple Mobile Robots

**DOI:** 10.3390/s100403261

**Published:** 2010-04-01

**Authors:** Cristina Losada, Manuel Mazo, Sira Palazuelos, Daniel Pizarro, Marta Marrón

**Affiliations:** Electronics Department, University of Alcalá, Campus Universitario s/n, 28805, Alcalá de Henares, Madrid. Spain. E-Mails: mazo@depeca.uah.es (M.M.); sira@depeca.uah.es (S.P.); pizarro@depeca.uah.es (D.P.); marta@depeca.uah.es (M.M)

**Keywords:** multi-camera sensor, intelligent space, motion segmentation, 3D positioning, mobile robots

## Abstract

This paper presents a method for obtaining the motion segmentation and 3D localization of multiple mobile robots in an intelligent space using a multi-camera sensor system. The set of calibrated and synchronized cameras are placed in fixed positions within the environment (intelligent space). The proposed algorithm for motion segmentation and 3D localization is based on the minimization of an objective function. This function includes information from all the cameras, and it does not rely on previous knowledge or invasive landmarks on board the robots. The proposed objective function depends on three groups of variables: the segmentation boundaries, the motion parameters and the depth. For the objective function minimization, we use a greedy iterative algorithm with three steps that, after initialization of segmentation boundaries and depth, are repeated until convergence.

## 1. Introduction

A common problem in the field of autonomous robots is how to obtain the position and orientation of the robots within the environment with sufficient accuracy. Several methods have been developed to carry out this task. The localization methods can be classified into two groups: those that require sensors onboard the robots [1] and those that incorporate sensors within the work environment [2].

Although the use of sensors within the environment requires the installation of an infrastructure of sensors and processing nodes, it presents several advantages, it allows reducing the complexity of the electronic onboard the robots and facilitates simultaneous navigation of multiple mobile robots within the same environment without increasing the complexity of the infrastructure. Moreover, the information obtained from the robots movement is more complete, thereby it is possible to obtain information about the position of all of the robots, facilitating cooperation between them. This alternative includes “intelligent environments” [3,4] characterized by the use of an array of sensors located in fixed positions and distributed strategically to cover the entire field of movement of the robots. The information provided by the sensors should allow the localization of the robots and other mobile objects accurately.

The sensor system in this work is based on an array of calibrated and synchronized cameras. There are several methods to locate mobile robots using an external camera array. The most significant approaches can be divided into two groups. The first group includes those works that make use of strong prior knowledge by using artificial landmarks attached to the robots [5,6]. The second group includes the works that use the natural appearance of the robots and the camera geometry to obtain the positions [2]. Intelligent spaces have a wide range of applications, especially in indoor environments such as homes, offices, hospital or industrial environments, where sensors and processing nodes are easy to install.

The proposal presented in this paper is included in the second group. It uses a set of calibrated cameras, placed in fixed positions within the environment to obtain the position of the robots and their orientation. This proposal does not rely on previous knowledge or invasive landmarks. Robots segmentation and position are obtained through the minimization of an objective function. There are many works that use an objective function [7,8]. However, the works in [7,8] present several disadvantages such as high computational cost or dependence on the initial values of the variables. Moreover, these methods are not robust because they use information from a single camera.

It is noteworthy that, although the proposal in this work has been evaluated in a small space (ISPACE-UAH), it can be easily extended to a larger number of rooms, corridors, *etc.* It allows covering a wider area, by adding more cameras to the environment and properly dimensioning the image processing hardware.

## 2. Multi-Camera Sensor System

The sensor system used in this work is based on a set of calibrated and synchronized cameras placed in fixed positions within the environment (Intelligent Space of University of Alcalá, ISPACE-UAH). These cameras are distributed strategically to cover the entire field of movement of the robots. As has been explained in the introduction, the use of sensors within the environment presents several advantages, it allows reducing the complexity of the electronic onboard the robots and facilitates simultaneous navigation of multiple mobile robots within the same environment without increasing the complexity of the infrastructure. Moreover, the information obtained from the movement of the robots is more complete, thereby it is possible to obtain information about the position of all of the robots, facilitating the cooperation between them.

### 2.1. Hardware Architecture

The hardware deployed in the ISPACE-UAH consists basically of a set of cameras with external trigger synchronization, a set of acquisition and processing nodes, mobile robots and a Local Area Network (LAN) infrastructure, that includes a wireless channel that the robots use to provide information from their internal sensors and to receive motion commands. All the cameras are built with a CCD sensor with a resolution of 640 × 480 and a size of 1/2” (8mm diagonal). The optical system is chosen with a focal length of 6.5 mm which gives about 45° of Field of View (FOV). Each camera is connected to a processing node through a Firewire (IEEE1394) local bus, which allows 25 fps RGB image acquisition speed and control of several camera parameters such as the exposure, gain or trigger mode.

The processing nodes are general purpose multi-core PC platforms with Firewire ports and Gigabit Ethernet hardware which allows them to connect to the LAN network. Each node has the capability of controlling and processing the information from one or several cameras. In the present paper each node is connected to a single camera.

The robotic platforms used in all experiments are provided by “Active Media Robotics”. More specifically, the model used is the P3-DX, which is a differential wheeled robot of dimensions 44.5 × 40 × 24.5 cm, equipped with low level controllers for each wheel, odometry systems and an embedded PC platform with IEEE 802.11 wireless network hardware.

### 2.2. Software Architecture

The software architecture chosen is a client-server system using common TCP/IP connections, where some servers (*i.e.,* processing nodes and robots internal PCs) receive commands and requests from a client (*i.e.,* computer or data storage device for batch tests).

**Figure 1.**
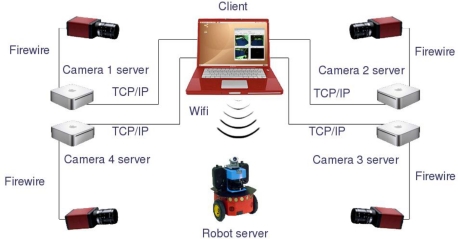
General diagram of the hardware/software architecture in the ISPACE-UAH.

Each processing node acts as a server that preprocesses the images and sends the results to the client platform. The preprocessing task of the servers consists of operations that can be clearly developed separately for each camera, such as image segmentation, image warping for computing occupancy grids, compression or filtering. The internal PC in each robot acts as a server which allows receiving control commands from a client and sending back the odometry readings obtained from its internal sensors. On the other hand, the client is in charge of performing data fusion using all information provided by the servers in order to achieve a certain task. In the case of the application proposed in this paper, the client receives robots odometry information, 3D occupancy grid representation of the scene and the client itself assures synchronization of the odometry values with the camera acquisition. In Figure 1, a general diagram of the proposed hardware/software architecture is shown.

### 2.3. Reference Systems in the Intelligent Space

**Figure 2.**
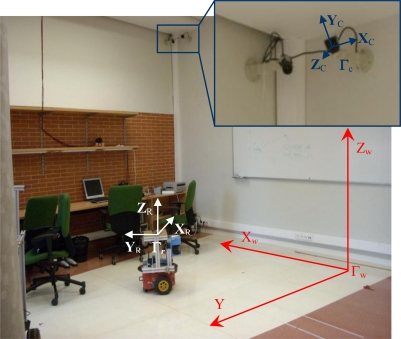
Reference systems in the intelligent space (ISPACE-UAH): World coordinate system (Γ*_w_*) in red color. Camera coordinate system (Γ*_ci_ i* = 1,2,…,*n_c_*) in blue color.

Before presenting the proposed algorithm for motion segmentation and 3D positioning of multiple mobile robots using an array of cameras, it is important to define the different coordinate systems used in this work. In the intelligent space, the 3D coordinates of a point **P** = (*X*, *Y*, *Z*)*^T^* can be expressed in different coordinate systems. There is a global reference system named “world coordinate system” and represented by Γ*_w_*. There is also a local reference system associated with each camera (Γ*_ci_*, *i* = 1,…,*n_c_*) whose origin is located in the center of projection. These coordinate systems are represented in Figure 2, where world coordinate system (Γ*_w_*) has been represented in red color and the coordinate systems associated to the cameras (Γ*_ci_*) have been represented in blue color.

**Figure 3.**
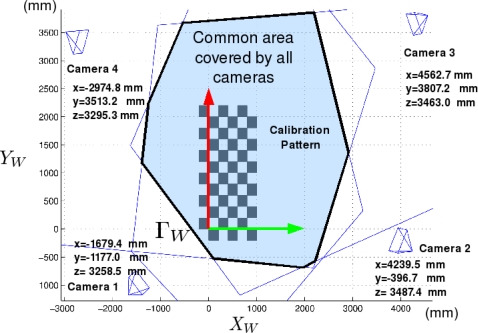
Spatial distribution of the cameras used in the experiments.

The cameras used in this work are placed in fixed positions within the environment (ISPACE-UAH). These cameras are distributed strategically to cover the entire field of movement of the robots. Figure 3 shows the spatial distribution, and the area covered by the cameras used in this work.

Cameras are modeled as pinhole cameras. This is a simple model that describes the mathematical relationship between the coordinates of a 3D point in the camera coordinate system (Γ*_c_*) and its projection onto the image plane in an ideal camera without lenses through the expressions in Equation (1) where *f_x_, f_y_* are the camera focal lengths along *x* and *y* axis:

(1) $$x=f_{x}\frac{X_{c}}{Z_{c}},\;y=f_{y}\frac{Y_{c}}{Z_{c}}$$

If the origin of the image coordinate system is not in the center of the image plane, the displacement (*s*_1_,*s*_2_) from the origin to the center of the image plane is included in the projection equations, obtaining the perspective projection Equation (2):

(2) $$x=f_{x}\frac{X_{c}}{Z_{c}}+s_{1},\;y=f_{y}\frac{Y_{c}}{Z_{c}}+s_{2}$$

These equations can be expressed using homogeneous coordinates, as shown in Equation (3):

(3) $$\left(\begin{array}{c} x\\ y\\ 1 \end{array}\right)=\left(\begin{array}{ccc} f_{x} & 0 & s_{1}\\ 0 & f_{y} & s_{2}\\ 0 & 0 & 1 \end{array}\right)\left(\begin{array}{c} X_{c}\\ Y_{c}\\ Z_{c} \end{array}\right)$$

**Figure 4.**
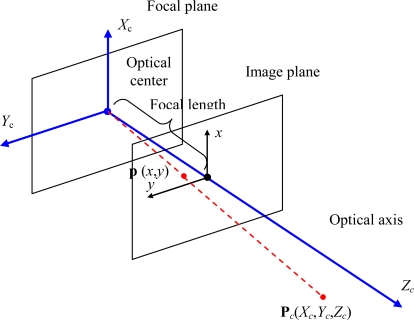
Geometric model of a pinhole camera. In this model the optical center coincides with the origin of the camera coordinate system (Γ*_c_*) represented in blue color. The image reference system (*x*,*y*) is drawn in black color.

The geometry related to the mapping of a pinhole camera is illustrated in the Figure 4.

## 3. Algorithm for motion segmentation and positioning

Using the work of Sekkati and Mitiche [7] as a starting point, in this work motion segmentation and 3D localization are obtained through the minimization of an objective function. The objective function proposed in [7] [and shown in Equation (4)] depends on three groups of variables: a set of curves that defines the mobile robot segmentation boundaries in the image plane ${\left\{\gamma_{k}\right\}}_{k=1}^{N-1}$, the components of linear and angular velocity of each robot ${\left\{v_{ck}\right\}}_{k=1}^{N}$, ${\left\{\omega_{\mathrm{ck}}\right\}}_{k=1}^{N}$ and the depth. In Equation (4) λ and μ are positive, real constants. These constants weight the contribution of each term to the objective function:

(4) $$E\left[{\left\{\gamma_{k}\right\}}_{k=1}^{N-1},{\left\{v_{\mathrm{ck}}\right\}}_{k=1}^{N},{\left\{\omega_{\mathrm{ck}}\right\}}_{k=1}^{N},Z\right]=\sum_{k=1}^{N}\left[\int_{\Omega_{k}}\psi_{k}^{2}(x)dx+\mu \int_{\Omega_{k}}g(||\nabla Z||)dx\right]+\sum_{k=1}^{N-1}\lambda \oint_{\gamma_{k}}ds,\;\begin{array}{c} \lambda ,\mu \in ℜ\\ \lambda ,\mu >0 \end{array}$$

As can be observed in Equation (4), the objective function proposed by Sekkati and Mitiche in [7] contains three different terms. The first term measures the conformity of the 3D interpretation within each region of segmentation to the image sequence spatiotemporal variations. This measure is given by the three-dimensional brightness constraint for rigid objects proposed in [7] and shown in Equation (5). The remaining two terms in Equation (4) are regularization terms, one for depth via a boundary preserving function (g(a)) and the other one for segmentation boundaries:

(5) $$I_{t}+s\frac{v_{c}}{Z_{c}}+{q\omega }_{c}=0$$

In Equation (5) **s** and **q** are two vectors that depend on the image spatiotemporal derivatives [*I_x_*, *I_y_*, *I_t_*], the coordinates of each point in the image plane (*x*, *y*) and the focal lengths *f_x_*, *f_y_*:

$$s={\left(\begin{array}{c} f_{x}I_{x}\\ f_{y}I_{y}\\ -\left(x-s_{1}\right)I_{x}-\left(y-s_{2}\right)I_{y} \end{array}\right)}^{T}\;q={\left(\begin{array}{c} -f_{y}I_{y}-\frac{y-s_{2}}{f_{y}}\left(\left(x-s_{1}\right)I_{x}+\left(y-s_{2}\right)I_{y}\right)\\ -f_{x}I_{x}-\frac{x-s_{1}}{f_{x}}\left(\left(x-s_{1}\right)I_{x}+\left(y-s_{2}\right)I_{y}\right)\\ -\frac{f_{x}}{f_{y}}\left(y-s_{2}\right)I_{x}+\frac{f_{y}}{f_{x}}\left(x-s_{1}\right)I_{y} \end{array}\right)}^{T}$$

In [7], the minimization of the objective function (4) is carried out using a greedy algorithm which consists of three iterated steps. After the initialization of the segmentation boundaries and depth, the three steps are repeated until the convergence of the algorithm. In each step, two of the three groups of variables are fixed, and the equation is solved for the remaining one. After minimization, motion segmentation of the mobile robots is obtained. However, proposal of [7] presents several disadvantages such as high computational cost, or dependence on the initial values of the variables (segmentation boundaries and depth). Moreover, this method is not robust, and it does not allow obtaining 3D position of the mobile robots because it uses information from a single camera.

Since there are multiple cameras available in the intelligent space, we have proposed a new objective function that includes information of all the cameras. The minimization of the proposed function allows us to obtain both motion segmentation and 3D position of multiple mobile robots in an intelligent space. The use of multiple cameras increases notably the robustness of the system. It also improves the accuracy of the results (segmentation and 3D positioning).

In addition, the proposed solution allows segmenting and estimating the 3D position of the mobile robots even if they are not seen by some of the cameras. Even in the worst case, if all the cameras lose some of the robots, they can still be controlled by the intelligent space. In this case, the positions of the unseen robots are estimated through the measurements of the odometry sensors they have onboard.

### 3.1. 3D Brightness Constraint for a Multi-camera Sensor System

Before presenting the objective function for multiple cameras, it is necessary to describe the 3D brightness constraint for multiple cameras, that is a generalization of the 3D brightness constraint for a single camera presented in [7].

Let **P***_w_*=(*X_w_*, *Y_w_*, *Z_w_*)*^T^* be the 3D coordinates of point **P** on a mobile robot related to the world coordinate system Γ*_w_*. Let $v_{w}={\left(v_{w}^{x}\;v_{w}^{y}\;v_{w}^{z}\right)}^{T}$ and $\omega_{w}={\left(\omega_{w}^{x}\;\omega_{w}^{y}\;\omega_{w}^{z}\right)}^{T}$ be, respectively, the components of the linear and angular velocity of the robot motion in Γ*_w_*. Then, the velocity of **P**, relative to Γ*_w_*, is given by Equation (6):

(6) $${\dot{P}}_{w}={\left(\begin{array}{ccc} {\dot{X}}_{w} & {\dot{Y}}_{w} & {\dot{Z}}_{w} \end{array}\right)}^{T}=v_{w}+\omega_{w}\times P_{w}$$

In the same way, if **P***_c_*=(*X_c_*, *Y_c_*, *Z_c_*)*^T^* are the coordinates of **P** relative to Γ*_c_* and $v_{c}={\left(v_{c}^{x}\;v_{c}^{y}\;v_{c}^{z}\right)}^{T}$ and $\omega_{c}={\left(\omega_{c}^{x}\;\omega_{c}^{y}\;\omega_{c}^{z}\right)}^{T}$ are the components of the linear and angular velocity of the robot motion in Γ*_c_*. The velocity of **P** relative to Γ*_c_* is given by Equation (7):

(7) $${\dot{P}}_{c}={\left(\begin{array}{ccc} {\dot{X}}_{c} & {\dot{Y}}_{c} & {\dot{Z}}_{c} \end{array}\right)}^{T}=v_{c}+\omega_{c}\times P_{c}$$

Let **R***_wc_* be the (3 × 3) rotation matrix and **T***_wc_* the (1 × 3) translation vector which represent the coordinate transformation from the world coordinate system (Γ*_w_*) to the camera coordinate system (Γ*_c_*). The coordinate transformation is carried out using the expression in Equation (8).

(8) $$P_{c}=R_{\mathrm{wc}}P_{w}+T_{\mathrm{wc}}$$

Differentiating the Equation (8) with respect to time, and substituting the expressions of the velocities in Γ*_w_* (Equation (6)) and Γ*_c_* (Equation (7)), Equation (9) is obtained:

(9) $$v_{c}+\omega_{c}\times P_{c}=R_{\mathrm{wc}}\left(v_{w}+\omega_{w}\times P_{w}\right)$$

Taking into account that cross product **ω** × **P** can be expressed as a scalar product **ω̂** · **P**, where **ω̂** is the following antisymmetric matrix:

$$\hat{\omega }=\left(\begin{array}{ccc} 0 & -\omega^{z} & \omega^{y}\\ \omega^{z} & 0 & -\omega^{x}\\ -\omega^{y} & \omega^{x} & 0 \end{array}\right)$$

Equation (9) can be rewritten to obtain Equation (10), where the components of linear and angular velocities in Γ*_c_* (**v***_c_*, **ω***_c_*) are expressed as a function of the components of velocity in Γ*_w_* (**v***_w_*, **ω***_w_*) and the transformation matrices (**R***_wc_*, **T***_wc_*):

(10) $$\begin{array}{c} v_{c}=R_{\mathrm{wc}}v_{w}-R_{\mathrm{wc}}\omega_{w}R_{\mathrm{wc}}^{T}T_{\mathrm{wc}}\\ \omega_{c}=\mathrm{adj}\left(R_{\mathrm{wc}}\right)\omega_{w} \end{array}$$

Let (*x*, *y*) be the coordinates of the projection of a point **P** on the image plane, the derivative of the perspective projection equations (Equation (2)) with respect to time, and the subsequent substitution of the expression of the velocity components of **P** in Γ*_c_* allows us to obtain the following equations for motion components in the image plane (*ẋ*,*ẏ*):

(11) $$\dot{x}=\frac{1}{Z_{c}}\left(f_{x}R_{\mathrm{wc}}^{1}-xR_{\mathrm{wc}}^{3}\right)v_{w}+q_{u}\mathrm{adj}\left(R_{\mathrm{wc}}\right)\omega_{w}$$

(12) $$\dot{y}=\frac{1}{Z_{c}}\left(f_{y}R_{\mathrm{wc}}^{2}-yR_{\mathrm{wc}}^{3}\right)v_{w}+q_{v}\mathrm{adj}\left(R_{\mathrm{wc}}\right)\omega_{w}$$

where $R_{\mathrm{wc}}^{i}$ is the *i*-th row in the rotation matrix from Γ*_w_* to Γ*_c_* (**R***_wc_*) and **q***_u_*, **q***_v_* are the following vectors:

$$\begin{array}{c} q_{u}=\left[x\left(\frac{t_{\mathrm{wc}}^{y}}{Z_{c}}-\frac{y}{f_{y}}\right)\;\left(f_{x}+\frac{x^{2}}{f_{x}}-\frac{1}{Z_{c}}\left(f_{x}t_{\mathrm{wc}}^{z}+xt_{\mathrm{wc}}^{x}\right)\right)\;f_{x}\left(\frac{t_{\mathrm{wc}}^{y}}{Z_{c}}-\frac{y}{f_{y}}\right)\right]\\ q_{v}=-\left[\left(f_{y}+\frac{y^{2}}{f_{y}}-\frac{1}{Z_{c}}\left(f_{y}t_{\mathrm{wc}}^{z}+yt_{\mathrm{wc}}^{y}\right)\right)\;y\left(\frac{t_{\mathrm{wc}}^{x}}{Z_{c}}-\frac{x}{f_{x}}\right)\;f_{y}\left(\frac{t_{\mathrm{wc}}^{x}}{Z_{c}}-\frac{x}{f_{x}}\right)\right] \end{array}$$

The substitution of velocity components in the image plane (*ẋ*,*ẏ*) in the well known brightness constraint (*I_x_ẋ* + *I_y_ẏ* + *I_t_*=0) allows to obtain a 3D brightness constraint for rigid objects in terms of the linear and angular velocity components in Γ*_w_* (**v***_w_* and **ω***_w_*). This constraint is shown in Equation (13):

(13) $$\Psi_{k}(x)=I_{t}+s\cdot R_{\mathrm{wc}}\frac{v_{w}}{Z_{c}}+q\cdot \mathrm{adj}\left(R_{\mathrm{wc}}\right)\omega_{w}+T_{\mathrm{wc}}^{T}r\cdot \mathrm{adj}\left(R_{\mathrm{wc}}\right)\frac{\omega_{w}}{Z_{c}}=0$$

where the matrices **s, q** and **r** in Equation (13) are given, respectively, by equations (14), (15) and (16):

(14) $$s=\left(\begin{array}{ccc} f_{x}I_{x} & f_{y}I_{y} & -\left(xI_{x}+yI_{y}\right) \end{array}\right)$$

(15) $$q=\left(-f_{v}I_{y}-\frac{y}{f_{v}}\left(x\cdot I_{x}+y\cdot I_{y}\right)\;f_{u}I_{x}+\frac{x}{f_{u}}\left(x\cdot I_{x}+y\cdot I_{y}\right)-\frac{f_{u}}{f_{v}}y\cdot I_{x}+\frac{f_{v}}{f_{u}}x\cdot I_{y}\right)$$

(16) $$r=\left(\begin{array}{ccc} 0 & -\left(xI_{x}+yI_{y}\right) & -f_{v}I_{y}\\ \left(xI_{x}+yI_{y}\right) & 0 & f_{u}I_{x}\\ f_{v}I_{y} & -f_{u}I_{x} & 0 \end{array}\right)$$

3D brightness constraint in Equation (13) must be satisfied in all of the *n_c_* cameras. Knowing it, we define a new 3D brightness constraint for rigid objects which includes all the information provided by the *n_c_* cameras available in the intelligent space [Equation (17)]:

(17) $$\psi_{\mathrm{ki}}(x)=I_{\mathrm{ti}}+s_{i}\cdot R_{\mathrm{wci}}\cdot \frac{v_{\mathrm{wk}}}{Z_{\mathrm{ci}}}+q_{i}\cdot \mathrm{adj}\left(R_{\mathrm{wci}}\right)\omega_{\mathrm{wk}}+t_{\mathrm{wci}}^{T}r_{i}\cdot \mathrm{adj}\left(R_{\mathrm{wci}}\right)\frac{\omega_{\mathrm{wk}}}{Z_{\mathrm{ci}}}\;\begin{array}{c} k=1,2,\ldots ,N\\ i=1,2,\ldots ,n_{c} \end{array}$$

Constraint in Equation (17) is defined for each region, in each camera. If there are *N*−1 robots in a scene, the scene is divided into *N* regions (region *N* corresponds to the background). We have added two subscripts to denote a region: subscript *k* (*k*=1,2,…,*N*), which indicates the region in each image, and subscript *i* (*i*=1,2,…,*n_c_*) which indicates the camera. It is worth pointing out that the components of the linear and angular velocity in the world coordinate system do not include the subscript *i* to indicate the camera because these velocities are equal for the *n_c_* cameras.

### 3.2. Objective Function for a Multi-camera Sensor System

The objective function for a multi-camera sensor system proposed in this work, Equation (18), depends on three groups of variables:

– A set of *N*−1 curves ${\left\{\gamma_{\mathrm{ki}}\right\}}_{k=1,\ldots ,N-1}^{i=1,\ldots ,n_{c}}$ that divide each image in *N* regions. These curves define the boundaries of the segmentation in the images acquired by each camera.
– The components of the linear and angular velocities ${\left\{v_{\mathrm{wk}}\right\}}_{k=1}^{N}$, ${\left\{\omega_{\mathrm{wk}}\right\}}_{k=1}^{N}$ of the (*N*−1) mobile robots and background. These velocities are related to the world reference system Γ*_w_* and are equal for the *n_c_* cameras.
– The depth (distance from each 3D point **P** to each camera). The value of depth in each point coincides with the *Z_ci_* coordinate of the point **P** related to the coordinate system of the camera *i* Γ*_ci_*:
  (18) $$E\left[{\left\{\gamma_{\mathrm{ki}}\right\}}_{k=1,\ldots ,N-1}^{i=1,\ldots ,n_{c}},{\left\{T_{\mathrm{wk}}\right\}}_{k=1}^{N},{\left\{\omega_{\mathrm{wk}}\right\}}_{k=1}^{N},{\left\{Z_{\mathrm{ci}}\right\}}_{i=1}^{n_{c}}\right]=\sum_{k=1}^{N}\sum_{i=1}^{n_{\mathrm{ck}}}\left[\int_{\Omega_{\mathrm{ki}}}\psi_{\mathrm{ki}}^{2}(x)dx+\mu \int_{\Omega_{\mathrm{ki}}}g\left(\left\|\nabla Z_{\mathrm{ci}}\right\|\right)dx\right]+\sum_{k=1}^{N-1}\sum_{i=1}^{n_{\mathrm{ck}}}\lambda \oint_{\gamma_{\mathrm{ki}}}\mathrm{ds}$$

In Equation (18), *ψ_ki_* is the 3D brightness constraint (defined in Equation (17)) for the pixels inside the curve *k* in the image acquired by the camera *i*; λ and μ are positive and real constants to weigh the contribution of the terms in the objective function (18) and ∇ = (∂*_x_*,∂*_y_*) is the spatial gradient operator.

As in the objective function for one camera (Equation (4)), the first term in (18) measures the conformity of 3D interpretation to the sequence spatiotemporal variations in each region through the 3D brightness constraint for a multi-camera sensor system. The second integral is a regularization term of smoothness of depth, and the third integral is a regularization term of the *N*−1 boundaries.

The objective function in Equation (18) includes information of all the cameras in the intelligent space. In this work, objective function minimization is carried out using a greedy algorithm that, after the initialization of the variables, consists of three iterative steps. Before the minimization, it is necessary to initialize the curves that define the contours of the segmentation and depth in the images acquired by each camera. Both, the initialization process and the minimization algorithm are explained below.

### 3.3. Curve and Depth Initialization

The initialization process is very important due to the high dependence of the results on the initial values of the variables. This process includes three different steps: in the first step, we obtain the initial curves. Since cameras are located in fixed positions within the intelligent space, the *N*−1 initial curves are obtained using GPCA (Generalized Principal Components Analysis) [9]. Then, the initial depth (relative to each camera coordinate system Γ*_ci_*) is obtained using Visual Hull 3D [10] which allows to obtain a 3D occupancy grid (composed by cubes with size Δh) in Γ*_w_* from the initial segmentation boundaries, that have been computed previously using GPCA. Finally, an extended version of the k-means algorithm is used to estimate the number of mobile robots in the scene. The three steps are described below.

As previously mentioned, GPCA [9] is used in this work to obtain a background model for each of the *n_c_* cameras. Background modeling is carried out from a set of background images that do not contain any mobile robot. Using GPCA we obtain two transformation matrices, **L***_ci_* and **R***_ci_*, for each camera. These matrices are calculated in each camera, and they represent the background model. Since the cameras are placed in fixed positions within the environment, the background modeling stage needs to be carried out only once, and it can be done off-line.

GPCA [9] is also used to initialize the segmentation boundaries by comparing each image to the background model. In this stage, each image is projected (Equation (19)) to the GPCA space using the matrices **L** and **R** (that have been obtained previously). After that, the image is reconstructed (Equation (20)). In these two equations **M** represents the mean of the *N_i_* images that have been used to obtain the background model:

(19) $$I_{T}=L^{T}(I-M)R$$

(20) $$I_{R}=LI_{T}R^{T}+M$$

Then, the reconstruction error is computed. This error is defined as the difference between the reconstructed (**I***_R_*) and the original (**I**) image and can be calculated subtracting the images pixel-to-pixel, but this approach is not robust against noise. Therefore, we define a set of pixels (window) around each pixel (with dimensions *q*x*q*) called **Φ***_wi_* in the original image an **Φ̂***_wi_* in the reconstructed image, and we obtain the reconstruction error for these windows, using Equation (21):

(21) $$\varepsilon_{\mathrm{wi}}=\left\|\Phi_{\mathrm{wi}}-{\hat{\Phi }}_{\mathrm{wi}}\right\|$$

Pixels whose reconstruction error (calculated using Equation (21)) is higher than a threshold are candidate to belong to a mobile robot, because in those pixels there is an important difference between the current image and the background model. The value of the threshold is very important. In this work we use an adaptive threshold [11].

**Figure 5.**
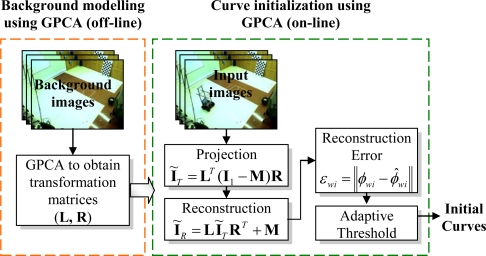
General block diagram of the proposed method for curve initialization using GPCA.

A block diagram including all the stages involved in curve initialization using GPCA is shown in Figure 5. All these stages have to be executed for each camera to obtain a set of initial curves ${\left\{\gamma_{\mathrm{ki}}\right\}}_{k=1,\ldots ,N-1}^{i=1,\ldots ,n_{c}}$.

**Figure 6.**
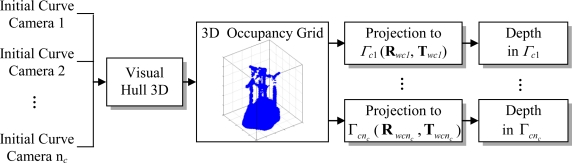
General block diagram of the proposed method for curve initialization using GPCA.

After curve initialization, Visual Hull 3D [10] is used to obtain a 3D occupancy grid (composed of cubes of size Δ*h*) in Γ*_w_* from the initial segmentation boundaries computed previously. The 3D coordinates of the occupied cell are projected from Γ*_w_* to each camera coordinate system Γ*_ci_* (*i*=1,…,*n_c_*) through the transformation matrices (**R***_wci_* and **T***_wci_*) to obtain a set of points on the mobile robots in Γ*_ci_*. This process provides an effective method for depth initialization in each camera. Figure 6 presents a block diagram including the main steps in the depth initialization process.

The algorithm used for motion segmentation and 3D positioning requires previous knowledge about the number of mobile robots. In order to estimate this value, we have included a clustering algorithm in the initialization process. In this stage, we project the coordinates of the occupied cell in the 3D occupancy grid obtained using Visual Hull 3D onto XY plane in Γ*_w_*. Then, we cluster the 2D data using an extended version of k-means [12]. This clustering algorithm allows us to obtain a good estimation of the number of robots in the scene, and a division of the initial curves in each image.

The use of GPCA and VH3D allows obtaining a set of initial values of the variables that are close to the real ones. Using these initial values, the objective function minimization converges after a few iterations. It is noteworthy that the reduction in the number of iterations until convergence with respect to the algorithms in [7,8] decreases notably the processing time of the proposed solutions.

### 3.4. Objective Function Minimization

After curve and depth initialization, objective function minimization is carried out. Because the proposed objective function (defined in Equation (18)) depends on three groups of variables, a greedy algorithm, which consists of three iterated steps, is used. In each step, two of the three groups of variables are fixed, and we solve the equation for the remaining one.

In the first step, we fix segmentation boundaries and depth in each Γ*_ci_*. So, the energy to minimize reduces to Equation (22):

(22) $$E\left({\left\{v_{\mathrm{wk}}\right\}}_{k=1}^{N},{\left\{\omega_{\mathrm{wk}}\right\}}_{k=1}^{N}\right)=\sum_{k=1}^{N}\sum_{i=1}^{n_{\mathrm{ck}}}\int_{\Omega_{\mathrm{ki}}}\psi_{\mathrm{ki}}^{2}(x)dx$$

Since the 3D brightness constraint for multiple cameras defined in Equation (17) depends linearly on the components of linear and angular velocity, 3D motion parameters in Γ*_w_* can be obtained solving the linear equation system shown in Equation (23):

(23) $$\left(\begin{array}{c} a_{k1}\left(x_{11}\right)\\ \vdots \\ a_{k1}\left(x_{p_{k1}1}\right)\\ a_{k2}\left(x_{12}\right)\\ \vdots \\ a_{kn_{c}}\left(x_{p_{kn_{c}n_{c}}}\right) \end{array}\right)\left(\begin{array}{c} v_{w}^{x}\\ v_{w}^{y}\\ v_{w}^{z}\\ \omega_{w}^{x}\\ \omega_{w}^{y}\\ \omega_{w}^{x} \end{array}\right)=\left(\begin{array}{c} -I_{t1}\left(x_{11}\right)\\ \vdots \\ -I_{t1}\left(x_{p_{k1}1}\right)\\ -I_{t2}\left(x_{12}\right)\\ \vdots \\ -I_{tn_{c}}\left(x_{p_{kn_{c}}n_{c}}\right) \end{array}\right)\;k=1,\ldots ,N$$

where: $a_{k}\left(x_{j}\right)=\left(\frac{S_{i1}}{Z_{\mathrm{ci}}},\frac{S_{i2}}{Z_{\mathrm{ci}}},\frac{S_{i3}}{Z_{\mathrm{ci}}},Q_{i1}+\frac{R_{i1}}{Z_{\mathrm{ci}}},Q_{i2}+\frac{R_{i2}}{Z_{\mathrm{ci}}},Q_{i3}+\frac{R_{i3}}{Z_{\mathrm{ci}}}\right)$.

In the second step, motion parameters and segmentation boundaries are fixed. In this step, the function to minimize is shown in Equation (24). In this function, χ*_ki_* is the characteristic function of region *k* in image *i* (Ω*_ki_*):

(24) $$E(Z)=\int_{\Omega }\sum_{K=1}^{N}\sum_{i=1}^{n_{\mathrm{ck}}}\left[\chi_{\mathrm{ki}}(x)\left(\psi_{\mathrm{ki}}^{2}(x)+\mu g\left(\left\|\nabla Z_{\mathrm{ci}}\right\|\right)\right)\right]dx$$

Given a set of contours ${\left\{\gamma_{\mathrm{ki}}\right\}}_{k=1,\ldots ,N-1}^{i=1,\ldots ,n_{c}}$ that divides each image in *N* regions (*N*−1 mobile robots and background), Equation (25) shows the descend equations for any region and for any camera. In this equation, τ indicates the algorithm execution time and *g*′ is the ordinary derivative of the boundary preserving function g. The boundary preserving function used in this work is a quadratic function (*g*(*a*) = *a*^2^). This is a simple function, but its effectiveness has been verified in several experiments, in which we have compared the results obtained using this quadratic function, and other boundary functions described in [13]:

(25) $$\frac{\partial Z_{\mathrm{ci}}}{\partial \tau }=\frac{2}{Z_{\mathrm{ci}}^{2}}\left(S_{i}v_{\mathrm{wk}}+R_{i}\omega_{\mathrm{wk}}\right)\psi_{\mathrm{ki}}+\mu \mathrm{div}\left(\frac{g^{\prime }\left(\left\|\nabla Z_{\mathrm{ci}}\right\|\right)}{\left\|\nabla Z_{\mathrm{ci}}\right\|}\nabla Z_{\mathrm{ci}}\right)\;\begin{array}{c} i=1,\ldots ,n_{\mathrm{ck}}\\ k=1,\ldots ,N \end{array}$$

The function to minimize in the third step is shown in Equation (27), where $\xi_{\mathrm{ki}}(x)=\psi_{\mathrm{ki}}^{2}(x)+\mu g\left(\left\|\nabla Z_{\mathrm{ci}}\right\|\right)$. This function is obtained after fixing depth and 3D motion parameters:

(26) $$E\left[{\left\{\gamma_{\mathrm{ki}}\right\}}_{k=1,\ldots ,N-1}^{i=1,\ldots ,n_{c}}\right]=\sum_{k=1}^{N}\sum_{i=1}^{n_{\mathrm{ck}}}\int_{\Omega_{\mathrm{ki}}}\xi_{k}(x)dx+\lambda \sum_{k=1}^{N-1}\sum_{i=1}^{n_{\mathrm{ck}}}\oint_{\gamma_{\mathrm{ki}}}\mathrm{ds}$$

As described in [7], for multiple region segmentation, the Euler-Lagrange descent equations shown in Equation (27) are obtained:

(27) $$\frac{\partial \gamma_{\mathrm{ki}}}{\partial \tau }(\tau )=-\left(\xi_{\mathrm{ki}}\left(\gamma_{\mathrm{ki}}\right)-\phi_{\mathrm{ki}}\left(\gamma_{\mathrm{ki}}\right)+\lambda \kappa_{\gamma_{\mathrm{ki}}}\left(\gamma_{\mathrm{ki}}\right)\right)\times n_{\mathrm{ki}}\left(\gamma_{\mathrm{ki}}\right)\;\begin{array}{c} i=1,\ldots ,n_{\mathrm{ck}}\\ k=1,\ldots ,N-1 \end{array}$$

In these equations, *κ*_*γ*_*ki*__ is the mean curvature and *n_ki_* is the exterior, unit, normal function of the curve *γ_ki_*. Functions φ*_ki_* are defined as: $\phi_{\mathrm{ki}}\left(\gamma_{\mathrm{ki}}\left(s\right)\right)=\underset{j\neq k}{\text{min}}\xi_{\mathrm{ji}}\left(\gamma_{\mathrm{ki}}\left(s\right)\right)$.

After initialization, the three described steps are repeated until the computed variables cease to evolve significantly.

## 4. Experimental Results

**Figure 7.**
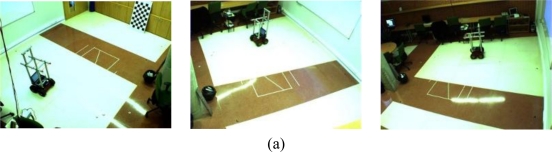

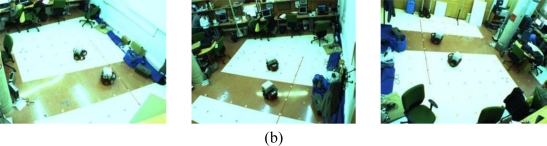
Images belonging to the test sequences, acquired by fixed cameras in the ISPACE-UAH. (a) Images belonging to the sequence 1 (b) Images belonging to the sequence 2.

In order to validate the proposed system, several experiments have been carried out in the ISPACE-UAH. In these experiments we have used two five-hundred image sequences. These sequences have been acquired using three of the four cameras in the ISPACE-UAH. Figure 7 shows one scene belonging to each sequence. As can be noticed in Figure 7, sequence 1 contains one robot whereas sequence 2 contains two mobile robots. The proposed algorithm for motion segmentation and 3D localization using a multi-camera sensor system has been used to obtain motion segmentation and 3D position for each couple of images in each sequence. All the experiments shown in this work have been carried out on Intel® core 2, 6600 with 2.4 GHz using Matlab.

**Figure 8.**
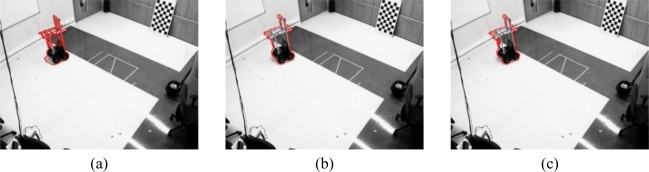
Boundaries of the segmentation obtained after the objective function minimization for one image belonging to the sequence 1 (Figure 7(a)). (a) Curves obtained using one camera (b) Curves obtained using two cameras (c) Curves obtained using three cameras.

**Figure 9.**
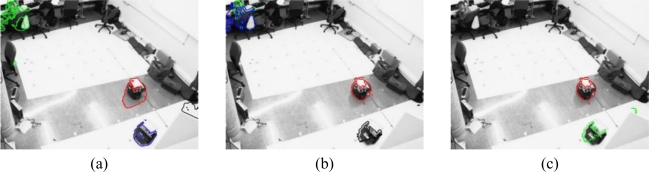
Boundaries of the segmentation obtained after the objective function minimization for one image belonging to the sequence 2 (Figure 7(b)). Each detected object is shown in a different colour (a) Curves obtained using one camera (b) Curves obtained using two cameras (c) Curves obtained using three cameras.

To start with the results, the boundaries of the motion segmentation in one image belonging to the sequence 1 [Figure 7(a)] and the sequence 2 [Figure 7(b)], respectively, are shown in Figure 8 and Figure 9 respectively.

Figure 8 shows the boundaries of the segmentation obtained for one image belonging to the sequence 1 [Figure 7(a)] that contains only one robot. In this figure we can observe that the result of the motion segmentation is similar regardless of the number of cameras considered. In all the images, the segmentation boundary is close to the real contour of the mobile robot in the image plane.

However, the boundaries obtained for an image belonging to the sequence 2 [Figure 7(b)] are notably different for 1, 2 or 3 cameras, as can be noticed in Figure 9. If the segmentation is carried out from the images acquired using one [Figure 9(a)] or two [Figure 9(b)] cameras, the person in the background of the scene is considered as a mobile robot but, if the images from three cameras are used, this person is not detected.

The computational time depends on both, the number of cameras and the number of robots detected in the scene. If the number of detected robots remains constant (as in sequence 1, where only one robot is detected for 1, 2 or 3 cameras) the processing time increases with the number of cameras. It can be observed in Table 1, where the average value of the computation time of each couple of images in the image sequences 1 and 2 is shown. In Table 1 we can observe that, for the images belonging to the sequence 1 (with only 1 robot) computation time increases with the number of cameras.

On the other hand, the number of objects detected as mobile robots has a bigger impact in the computation time than the number of cameras, as can be noticed in Table 1. The sequence 1, used to obtain the results in Table 1, contains only one robot whereas the sequence 2 includes two robots. Comparing the results obtained for the sequence 1 and the sequence 2, it can be noticed that, regardless of the number of cameras, the processing time obtained for the sequence 2 (with two robots) is higher than the processing time obtained for the sequence 1 (including only one robot). Moreover, in case of the sequence 2, the computation time using two cameras is bigger than using three cameras. The reason is that the number of objects that have been segmented with 2 cameras is bigger.

**Figure 10.**
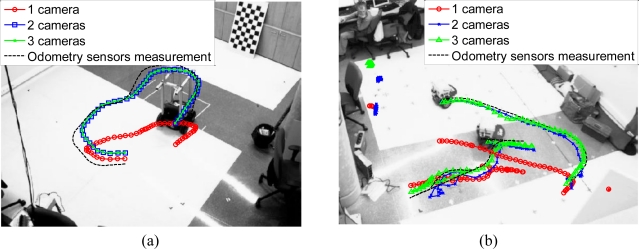
3D trajectory estimated by the algorithm and measured by the odometry sensors on board the robots projected onto the image plane (a) Image belonging to the sequence 1 (b) Image belonging to the sequence 2.

With regard to 3D positioning, Figure 10 shows the projection, onto the image plane, of the 3D trajectory of the mobile robot estimated by the algorithm (using 1, 2 and 3 cameras) and measured by the odometry sensors on board the robots. The represented trajectory has been calculated using 250 images belonging to each sequence.

The trajectories shown in Figure 10 are obtained by projecting the estimated trajectory in Γ*_w_*, obtained using the proposed algorithm, onto the image plane of the camera 1.

**Figure 11.**
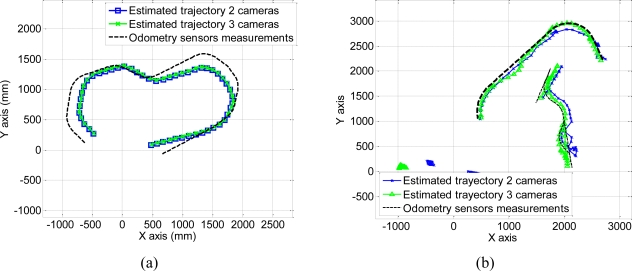
3D trajectory estimated by the algorithm and measured by the odometry sensors on board the robots on the *X_w_*, *Y_w_* plane (a) Sequence 1 (b) Sequence 2.

These trajectories obtained using 250 images belonging to each sequence can also be represented in the world coordinate system. The coordinates of the centroid of the points belonging to each robot are projected onto the plane (*X_w_*, *Y_w_*) in Γ*_w_* to obtain the 3D position. The result of this projection for a 250 images belonging to each sequence is shown in Figure 11. In this figure, we have represented the estimated trajectory obtained using 2 and 3 cameras.

As can be observed in Figure 10 and Figure 11, the estimated trajectories are closer to the measurements of the odometry sensors as the number of cameras increases. This fact can also be observed in the positioning error calculated as the difference between the estimated and the measured positions along *X_w_* and *Y_w_* axis, using Equation (28):

(28) $$\varepsilon_{p}=\sqrt{\varepsilon_{\mathrm{px}}^{2}+\varepsilon_{\mathrm{py}}^{2}}$$

**Figure 12.**
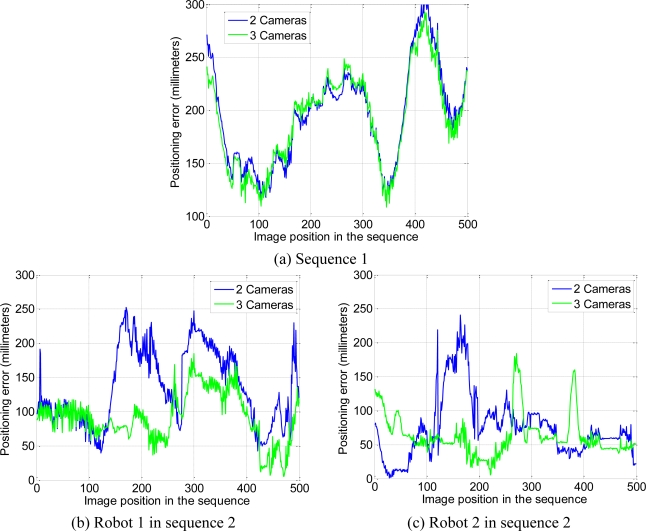
Positioning error (in millimetres) of the mobile robots, calculated using Equation (28) for 2 and 3 cameras. (a) Robot in the image sequence 1 (b) Robot 1 in the image sequence 2 (c) Robot 2 in the image sequence 2.

The positioning error, calculated for 500 images belonging to each sequence, has been represented in Figure 12. It is worth highlighting that the wheels of the robot in sequence 1 tend to skid on the floor. This is the reason why, in some positions, the difference between the estimated position and the measured one is high.

As can be observed in Figure 12, using the multi-camera sensor system with 2 or 3 cameras the positioning error is lower than 300 millimeters. It can also be observed in Table 2, where the average value of the positioning error for 500 images represented in Figure 12 is shown. Moreover, the positioning error reduces as the number of cameras increases. This reduction is more important in the sequence 2. It is because sequence 2 is more complex than sequence 1 and the addition of more cameras allows removing the points that do not belong to mobile robots and dealing with robot occlusions.

Finally, it is noteworthy that although we have obtained better results using the images acquired by three cameras, two cameras are enough to obtain suitable 3D positions. For this reason, we can conclude that the proposal in this paper can work properly even if one of the three cameras looses track of one robot. Even in the worst case, if all the cameras lose some of the robots, they can still be controlled by the intelligent space. In this case, the positions of the unseen robots are estimated through the measurements of the odometry sensors they have onboard.

## 5. Conclusions

A method for obtaining the motion segmentation and 3D localization of multiple mobile robots in an intelligent space using a multi-camera sensor system has been presented. The set of calibrated and synchronized cameras are placed in fixed positions within the environment (in our case, the ISPACE-UAH). Motion segmentation and 3D position of the mobile robots are obtained through the minimization of an objective function that incorporates information from the multi-camera sensor. The proposed objective function has a high dependence on the initial values of the curves and depth. In this sense, the use of GPCA allows obtaining a set of curves that are close to the real contours of the mobile robots. Moreover, Visual Hull 3D allows us to relate the information from all the cameras, providing an effective method for depth initialization. The proposed initialization method guarantees that the minimization algorithm converges after a few iterations. The reduction in the number of iterations also decreases the processing time against other similar works.

Several experimental tests have been carried out in the ISPACE-UAH and the obtained results validate the proposal presented in this paper. It has been demonstrated that the use of a multi-camera sensor increases significantly the accuracy of the 3D localization of the mobile robots against the use of a single camera. It has also been proved that, the positioning error decreases as the number of cameras increases. In any case, using a multi-camera sensor, the positioning error is lower than 300 millimeters. With regard to the processing time, it depends on both, the number of cameras and the number of robots detected in the scene, having the second factor a bigger impact. In fact, the processing time can be reduced if the number of cameras is increased, because the noise measurements (that do not belong to mobile robots) are reduced when the number of cameras is increased.

Regarding to the future work, the most immediate task is the implementation of the whole system in real time. Currently the system is working in a small space (ISPACE-UAH). It will be extended, in order to cover a wider area, by adding more cameras to the environment and properly re-dimensioning the image processing hardware. This line of future work has a special interest towards its installation in buildings with multiple rooms.

## Figures and Tables

**Table 1.** Average value of the computation time (in seconds) of each couple of the 500 images belonging to each test sequence for 1, 2 and 3 cameras.

	**Sequence 1 (contains one robot)**			**Sequence 2 (contains two robots)**		
	**1 camera**	**2 cameras**	**3 cameras**	**1 camera**	**2 cameras**	**3 cameras**
**Initialization**	0.2910	2.8353	3.3234	0.3410	5.1390	4.0753
**Minimization**	0.8758	2.8247	4.1588	2.7273	9.8419	6.9925
**Total**	1.1668	5.6600	7.4822	3.0683	14.9810	11.0678

**Table 2.** Average value of the value of the positioning error (mm) obtained using 500 images belonging to each test sequence.

	**Sequence 1**	**Sequence 2**	
	Robot1	Robot 1	Robot 2
**1 Camera**	1001.5683	371.8227	769.7783
**2 Cameras**	194.8882	136.1451	75.3317
**3 Cameras**	191.7257	91.5264	63.2390
